# Imaging the pathophysiology of major depressive disorder - from localist models to circuit-based analysis

**DOI:** 10.1186/2045-5380-4-5

**Published:** 2014-03-07

**Authors:** Michael T Treadway, Diego A Pizzagalli

**Affiliations:** 1 Center for Depression Anxiety and Stress Research, McLean Hospital/Harvard Medical School, 115 Mill Street, Belmont, MA 02478, USA

**Keywords:** Major Depression, Neuroimaging, PET, MRI, Serotonin, Dopamine, MRS, Glutamate, GABA, Inflammation

## Abstract

The neuroimaging literature of Major Depressive Disorder (MDD) has grown substantially over the last several decades, facilitating great advances in the identification of specific brain regions, neurotransmitter systems and networks associated with depressive illness. Despite this progress, fundamental questions remain about the pathophysiology and etiology of MDD. More importantly, this body of work has yet to directly influence clinical practice. It has long been a goal for the fields of clinical psychology and psychiatry to have a means of making objective diagnoses of mental disorders. Frustratingly little movement has been achieved on this front, however, and the 'gold-standard’ of diagnostic validity and reliability remains expert consensus. In light of this challenge, the focus of the current review is to provide a critical summary of key findings from different neuroimaging approaches in MDD research, including structural, functional and neurochemical imaging studies. Following this summary, we discuss some of the current conceptual obstacles to better understanding the pathophysiology of depression, and conclude with recommendations for future neuroimaging research.

## Introduction

The neuroimaging literature of Major Depressive Disorder (MDD) has exploded in recent years, with the current pace of research including over 250 new articles listed each year in PubMed alone. A substantial majority of these studies have been focused on identifying putative biological and neural variables that differentiate individuals with MDD from psychiatrically healthy controls. This program of research has been successful in demonstrating a large number of abnormalities in MDD samples, including alterations across measures of brain structure and function; endocrine, immune and neurotransmitter systems; and large-scale network organization. Despite this progress, however, fundamental questions remain about the pathophysiology and etiology of MDD as well as the strengths and pitfalls of neuroimaging methodologies in attempting to answer them.

Even more importantly, this body of work has yet to influence clinical practice in any substantive way. A longstanding goal of clinical psychology and biological psychiatry research has been the development of objective tests for diagnosing mental disorders. Frustratingly little progress has been made on this front, however, and the 'gold-standard’ of diagnostic validity and reliability remains expert consensus, a practice that is essentially unchanged from Meehl’s day [1]. Despite our capacity to measure an astonishing array of biological signals in MDD patients, we have yet to find a single measure - or a combination of variables - that tracks symptom expression with the requisite specificity and sensitivity to be reliably meaningful in the clinic.

It is against this backdrop that we present the current review. First, we provide a (non-exhaustive) summary of the major findings that have emerged from different neuroimaging approaches. This includes a review of structural, functional, neurochemical, neuroendocrine and neuroimmune imaging studies in MDD. Following this summary, we discuss some of the current conceptual obstacles to better understanding the pathophysiology of depression, and present the use of circuit-based analysis as a methodological path forward.

## Review

### Neuroimaging and the pathophysiology of MDD

#### Morphometric neuroimaging studies

A large number of studies in MDD patients to date have identified structural alterations across multiple tissue classes. These findings have been summarized using meta-analytic approaches reporting on structural alterations observed using regions-of-interest (ROI) tracing-based methods [2,3], voxel-based methods (VBM) [4], post-mortem tissue analysis [5], and diffusion tensor imaging of white-matter integrity [6]. Tracer-based methods have provided especially strong evidence for reduced hippocampal volume and enlarged ventricles in MDD [3]. These results have been recapitulated by voxel-based methods, which additionally implicate a more distributed network of structural alterations associated with MDD, including the anterior cingulate cortex (ACC), medial prefrontal cortex (mPFC), orbitofrontal cortex (OFC), dorsolateral prefrontal cortex (dlPFC), the striatum, and the amygdala. A limitation of these findings is that they are mostly drawn from cross-sectional designs. Therefore, it is difficult to know whether such structural differences represent a biological diathesis, a compensatory adaptation, or a consequence of the illness.

A handful of longitudinal studies have addressed this significant limitation by investigating structural changes as a function of depressive state and treatment outcome. \For some regions, including the hippocampus and medial prefrontal areas, several studies have suggested that grey-matter volume may decline monotonically over multiple depressive episodes [7,8]. In contrast, the amygdala may become enlarged prior to a first depressive episode [9]. Structural integrity of these regions has also been found to partially predict symptom remission. Specifically, hippocampal volumes have been found to prospectively correlate with treatment outcome at both one- and three-year follow-ups [10,11], and longitudinal studies have found that decreased hippocampal volumes were partially restored following successful treatment or spontaneous remission [11-13]. Importantly, similar morphometric changes in these regions have also been associated with high levels of trait negative affect in non-depressed individuals who have an elevated polygenic risk profile for developing MDD. This has been observed using both genome-wide analysis [14] and examinations of non-depressed individuals with a family history of MDD [15,16], consistent with the hypothesis that these structural decreases likely reflect an endophenotype marker [17].

Taken together, structural imaging studies have found robust evidence for group-level differences in grey-matter volume across cortical and sub-cortical areas. Longitudinal studies provide the strongest evidence linking these changes to the onset and remission of a depressive state, suggesting that they are either causally involved in MDD or that they at least meaningfully fluctuate with illness progression. Future longitudinal work, especially with prospective-cohort designs, will help further elucidate the role of these morphometric alterations in the etiopathophysiology of MDD.

#### Functional neuroimaging studies

**Figure 1:**
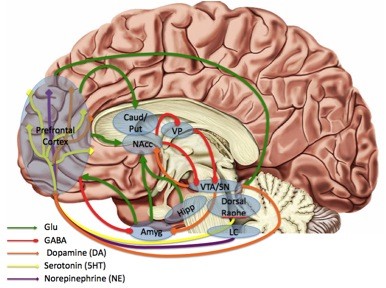
**Regions, transmitters and circuits implicated in the pathology of major depressive disorder (MDD) by human neuroimaging studies.** Past studies have identified alterations in monoamine levels and receptor availability as well as alterations in glutamate and GABA. These neurotransmitter systems participate in larger circuits involved in the experience and regulation of emotion, responses to stress, and processing of rewards. Note: placement of structure labels is approximate. Amyg = amygdala; Caud = Caudate; GABA = GABAergic projections; Glu = glutamatergic projections; Hipp = hippocampus; NAcc = nucleus accumbens; Put = Putamen; SN = substantia nigra; VP = ventral pallidum; VTA = ventral tegmental area. Republished with permission from Treadway and Zald [49].

Functional imaging studies of MDD have also grown substantially over the last two decades, with a wide variety of functional domains probed using a large number of tasks. We will therefore limit our focus to two general domains that have been most frequently examined in depression studies: (1) emotion processing and regulation, and (2) reward processing (Figure 1). For a more comprehensive discussion of other aspects of the functional neuroimaging literature in major depression, we refer readers to several excellent recent reviews and meta-analyses: [18-20].

##### Functional neuroimaging of emotion processing

Arguably the most common domain assessed by functional imaging studies of depression is responses to emotional stimuli. Examples include studies of responses to both explicit and implicit presentations of affect-laden stimuli [21-23], recruitment of cognitive control mechanisms required to gate out affective 'distractors’ during simple working memory and attention tasks [24,25], and deliberate top-down control of affective responses to positive and negative stimuli [26-29]. The most replicated result observed during passive presentation of emotional stimuli is a heightened responsivity in limbic regions - especially the amygdala - to negatively valenced stimuli in depressed individuals. For tasks that require subjects to efficiently 'gate-out’ affective content in order to better attend to non-emotional aspects of a task or stimulus, elevated limbic activity is often accompanied by hypo-activation in prefrontal areas, including aspects of ventromedial PFC, ventrolateral prefrontal cortex (vlPFC), ACC, and dlPFC. It is noteworthy that these same regions frequently exhibit volumetric abnormalities.

While prefrontal hypo-activations are commonly interpreted as evidence of a top-down control 'deficit’, it is unclear whether they reflect a local deficit in network recruitment or simply a failure to engage in the task as effectively as controls. Interestingly, when task performance is matched across depressed and non-depressed individuals, there is evidence for hyper-response in prefrontal areas [30,31], possibly indicating cortical inefficiency. In addition, the specificity of alterations in amygdalar and prefrontal networks to depression is unclear, as similar patterns are frequently observed in studies of anxiety, and only a few direct comparison studies have been conducted to date [32,33]. Future research is needed to further isolate the specific alterations in cortico-limbic responses to emotion in MDD, and to determine the extent to which these effects are specific to a depressed mood or rather represent a common mechanism associated with other forms of internalizing psychopathology.

In contrast to experimental paradigms that require either passive emotional processing or implicit emotion regulation in the form of attentional control, findings of studies of directed emotion regulation in MDD are highly variable. In healthy controls, down-regulation of negative emotion has been consistently associated with increased activation in medial and dlPFC areas and reduced activity in the amygdala [34]. These observations, combined with observations of impaired functional coupling between mPFC and amygdala during passive viewing of affective stimuli [35], led investigators to hypothesize that depressed patients would be less successful in reducing amygdala reactivity - and associated negative emotions - when explicitly regulating emotional responses to negative stimuli. Empirical support for this hypothesis, however, has been mixed. Only one study has reported that depressed patients experience more difficulty in decreasing sadness than controls [26], while others have found no differences [27-29]. These studies have also generally failed to observe impaired cortico-amygdala interactions during explicit emotion regulation in MDD. Consequently, these data suggest that emotion regulation deficits in MDD do not reflect a true inability to regulate emotion when explicitly directed to do so, at least not in the context of typical laboratory-based affective stimuli.

##### Functional neuroimaging of reward processing

Another primary area of functional neuroimaging research in MDD involves responses to rewarding stimuli. While early functional magnetic resonance imaging (fMRI) (and non-imaging) studies frequently operationalized reward in terms of the passive viewing or consumption of positively valenced stimuli (for example [36-38]), more recent work has increasingly emphasized constructs of reward anticipation [39-42], reinforcement learning [43,44] and motivation [45-47], which are psychologically and neurobiologically distinct. This shift has been motivated largely by the enhanced understanding of functional segregation of dopaminergic cortico-striatal systems in reward processing, which have been found to underlie anticipation, learning, and salience of rewards, rather than affective responses to them [48]. Indeed, reward-related symptoms are especially amenable to a translational neuroscience approach, given how well characterized reward-related pathways are by both preclinical and human neuroeconomic studies (see [49] for a longer discussion). The most common observation from this body of work is hypo-recruitment in MDD patients of striatal regions associated with reward salience, anticipation and learning, possibly reflecting alterations in the availability of pre-synaptic pools in dopaminergic afferents to striatal sub-regions [50-52] (see also discussion of dopamine imaging studies below). In addition, altered cross-talk between cortical and ventral striatal regions has been associated with rapid habituation to rewarding stimuli, which is also consistent with anhedonic presentation [53].

In sum, these studies highlight cortico-striatal pathways as critically involved in specific symptom domains of MDD. Of note, there is arguably greater consistency in studies of reward processing in MDD than of other cognitive processes. This may reflect the fact that reward processing studies have focused on a more homogeneous symptom domain and that the neurobiology of normative reward functioning is better understood.

#### Neurochemical imaging studies in MDD

The hypothesis that specific neurotransmitter systems represent a core pathology of mood disorders is among the oldest in biological psychiatry (see, for example, Schildkraut [54]). For most of modern psychiatric history, this line of work has emphasized alterations in monoamines, given early observations that administration of various monoamine-reducing drugs or pharmacological manipulations could induce depressive symptoms. It was only decades later, however, when the *in vivo* visualization of these signaling pathways could be achieved.

Currently, the two most widely used approaches to neurochemical imaging in psychiatric populations are Positron Emission Tomography (PET) and Magnetic Resonance Spectroscopy (MRS). A less commonly used technique is Single Photon Emission Computed Tomography (SPECT). Both PET and SPECT rely on the measurement of radioactive decay from an injected isotope as the basis of targeting the spatial distribution of a particular receptor or protein. In contrast, MRS takes advantage of the different magnetic resonance signatures associated with distinct molecular compounds, and can be useful for quantifying the availability of relatively abundant neurotransmitters such as glutamate (Glu) and γ-aminobutyric acid (GABA). Both of these methods have contributed to the study of pathophysiology in MDD, and are notable for both their positive and null findings. In this section, we review some of the primary neurotransmitter systems that have been investigated in MDD using these techniques.

##### Neurochemical imaging of serotonin systems in MDD

Interest in serotonin (5-HT) has been central to depression research over the last three decades, owing primarily to reported success of antidepressant pharmacotherapies that selectively target the serotonergic system in both humans and animal models. Evidence from preclinical studies further supports a role for serotonin in MDD symptoms, particularly those related to the processing of stress. Under normal conditions of wakefulness, serotonin neurons are tonically active [55] and the distribution of serotonergic tone is relatively even across most brain regions [56] - a pattern that has been found to support normal network functioning for a variety of cognitive and goal-directed behaviors. In contrast, exposure to stress can produce a surge in 5-HT signaling, which has been found to disrupt the emotion-regulatory functions of cortico-amygdalar networks [57]. Further, evidence suggests that medial prefrontal projections to serotonin-releasing neurons in the dorsal raphe play a crucial role in determining adaptive versus non-adaptive responses to stress [58,59]. Consequently, impaired serotonin signaling may be a substrate involved in stress vulnerability and a key risk factor in the development of MDD [60-62].

For these reasons, serotonin is among the most widely imaged neurochemical systems in MDD, with over 35 studies exploring group differences in the expression of serotonin receptor sub-types as well as the serotonin transporter (for recent reviews, see [63,64]). To date, however, results have been mixed, with investigators frequently reporting higher or lower serotonin receptor or transporter expression in MDD participants than in controls [63]. For example, of the 15 studies investigating expression of 5-HT_1A_ receptor in depressed patients relative to healthy controls, nine reported decreased expression in MDD, four reported increased expression, and two observed no change. Similar discrepancies have been observed for other proteins involved in 5-HT signaling pathways, including the 5-HT_2A_ receptor, 5-HT_1B_ receptor, and the serotonin transporter (SERT).

It is important to note that most of these studies are relatively small in size (between 9 and 22 MDD patients) and are, therefore, underpowered to explore within-sample relations between serotonin function and specific symptom dimensions. This is a potentially critical limitation, as the substantial heterogeneity of MDD is likely to be associated with divergent effects on neurotransmitter systems. In addition, most of these studies have not investigated the function of serotonin signaling systems, compared to baseline expression. Moreover, no longitudinal studies have been performed. Therefore, as with cross-sectional studies of structure or function, it is difficult to know whether 5HT abnormalities should be interpreted as a primary deficit, a downstream consequence, a risk factor, or a compensatory mechanism. What is clear, however, is that to the extent that the 5-HT system is involved in the etiopathophysiology, its effect size is modest, and likely dependent on interactions with numerous other systems.

##### Neurochemical imaging of catecholamine systems in MDD

Other monoamines that have long been associated with MDD are the catecholamines dopamine (DA) and norepinephrine (NE) [65,66]. DA is well established as being necessary for motivation, reward-based learning, and goal-directed behavior [48,67,68] and, therefore, is believed to be a substrate of reward-related symptoms such as anhedonia, fatigue, and anergia in psychiatric disorders [69,70]. Unlike 5-HT, which is relatively uniform in its distribution across the brain, DA expression is densest in the striatum, a key structure involved in valuation, decision-making and action.

Neuroimaging evidence for altered DA systems in MDD comes primarily from PET, SPECT and pharmacological challenge studies. This research has found that MDD is associated with changes in DA synthesis capacity as indexed by L-3,4-dihydroxyphenylalanine (L-DOPA) uptake [71], as well as changes in the regional distribution and availability of DA receptors, and the DA transporter (DAT). As with the 5-HT studies summarized above, however, imaging studies of DA systems have produced conflicting results. In PET and SPECT studies of DAT, MDD has been associated with both lower [72] and higher [73-75] binding potential in the striatum. Interestingly, all studies reporting DAT increases have used SPECT, which has much lower sensitivity than PET [76] and often employed tracers that have equal affinity for the SERT and DAT (for example, β-CIT) and thus do not allow conclusive interpretations. Moreover, post-mortem studies support the observation of reduced DAT expression [77].

Studies of DA receptor availability in MDD have also yielded mixed results. In some cases, increased striatal D2/D3 receptor binding has been shown to occur in heterogeneous depressed samples [78,79]. This increase in D2/D3 receptor availability appears to contradict animal data in which antidepressant responses are associated with increased D2-like binding in the striatum [80]. Other studies using medication-naïve or medication-free patients have failed to find group differences in striatal receptor binding [81,82], while one additional small study reported variable changes in D2-like binding following treatment with selective serotonin reuptake inhibitors (SSRIs) with patients who showed increased binding exhibiting more clinical improvement than those who did not [83]. With respect to the D1 receptor, fewer studies have examined this system given the lack of available ligands that reliably distinguish between the D1 and serotonin 5-HT_2A_ receptor, especially in extra-striatal areas where the receptor density of D1 and 5HT_2A_ is roughly equivalent. One study reported reduced D1 availability in left middle caudate [84], but this finding has not yet been replicated. Taken together, these studies suggest a possible role of D2-like receptors in downstream effects of antidepressant treatment, although the precise nature of the effect and how alterations in D2-like receptor availability may be related to DA function are unclear.

As with other conflicting reports in neuroimaging studies of MDD, part of the discrepancy across studies likely reflects the heterogeneity of the disorder. Supporting this assertion is the observation of slightly more consistent effects when MDD samples are selected on the basis of a particular symptom profile. For example, one study that restricted its MDD patient sample to individuals with anhedonic symptoms reported decreased DAT binding [85]. In addition, L-DOPA alterations in the striatum are present in depressed individuals with flat affect or psychomotor slowing, but not in depressed individuals without these symptoms [86,87]. Decreases in DA synthesis have also been observed in patients who develop depressive symptoms after undergoing IFN-α therapy [50]. This therapy stimulates inflammation signaling cascades, which have been found to disrupt DA synthesis, and may provide a link between elevated inflammation in MDD and specific symptoms related to perturbances of DA signaling, such as motivation and anhedonia [50,88]. Overall, these studies provide mixed evidence for general DA alterations in MDD, with additional evidence highlighting the importance of examining links between DA systems and specific symptoms in MDD, rather than in the disorder as a whole.

In contrast to DA, molecular imaging methods of NE signaling pathways have been much slower to develop. Currently, only studies of the NE transporter (NET) have been performed in MDD [89], with no studies examining NE receptors in MDD due to a lack of available ligands. Pharmacological functional imaging studies have also been used to indirectly explore effects of NE-increasing agents, though many of these studies have used drugs such as duloxetine, which simultaneously target both 5-HT and NE transporters. Duloxetine reduced connectivity within resting-state and task-positive networks, [90], and boosted ventral striatal responses during a reward task [91], while the NET-selective agent reboxetine increased thalamic dorsolateral prefrontal responsivity to emotional pictures [92,93]. While these studies provide promising leads, insufficient functional or molecular imaging work of NE function in the context of MDD is available, despite significant evidence for its role in the disorder [94].

##### Neurochemical imaging of glutamatergic and GABAergic systems in MDD

In recent years there has been substantial interest in the contribution of non-monoamine neurotransmitters to the pathophysiology of MDD, particularly the excitatory and inhibitory amino acid transmitters of glutamate (Glu) and GABA, respectively. At an intuitive level, the hypothesis that these systems would be implicated in depression holds significant appeal; the innervation of Glu- and GABA-releasing neurons vastly outnumbers all other neurotransmitter systems in the brain, making these two neurochemicals responsible for the bulk of information processing related to learning, cognition, memory, and decision-making [95]. When considering the scope of this diverse functional anatomy, it is difficult to imagine that Glu and GABA would not be directly, or at least indirectly, involved.

Evidence for alterations of Glu transmission in MDD have long been reported, but findings have been mixed, with increased Glu levels observed in plasma samples and post-mortem tissue as compared to decreased levels found in neuroimaging studies [96-98]. These discrepancies may be due in part to the multiple roles that Glu plays in the brain (for a more extended discussion, see [99]). A recent meta-analysis or MRS imaging studies found that MDD was associated with a substantial decrease in Glu levels within the mPFC/ACC [100], though it should be noted that not all studies were able to distinguish between Glu and glutamine, a common metabolite of astrocyte reuptake processes. Studies published after this meta-analysis provided additional evidence of reduced Glu concentration in the mPFC of MDD subjects [101-103], and similar alterations have also been detected in children with depressive symptoms [104] as well as remitted MDD subjects [102], raising the possibility that they constitute a trait-like vulnerability factor for MDD. Highlighting the clinical significance of these findings, among MDD subjects, increased pre-treatment Glu levels predicted better electroconvulsive therapy (ECT) response [103].

PET imaging studies of metabotropic Glu receptors have also revealed changes in Glu signaling pathways in MDD [105] and in relation to MDD symptoms [106]. The rapid antidepressant effects of ketamine, an N-methyl-D-aspartate (NMDA) partial agonist [107,108] further implicate the Glu pathway. Finally, aberrations in Glu signaling and Glu neurotoxicity have been associated with mPFC volumetric reductions discussed above [95]. In sum, while investigation of Glu dysfunction in MDD is relatively new, given the near ubiquitous distribution of Glu signaling throughout the brain, it is likely that many of the alterations in neural circuit function observed using fMRI studies partially reflect Glu-related pathology.

In contrast to Glu, studies of GABA are less frequent in MDD. GABA alterations have been documented in MDD [17,109], including reports of reduced GABA levels in plasma and cerebrospinal fluid [110-112], as well as specific GABA reductions in the mPFC as assessed with MRS [113,114]. Moreover, GABA function in this region has been suggested to play a critical role in mediating negative feedback of hypothalamic-pituitary-adrenal (HPA)-axis activity [115,116]. Thus, decreased GABAergic tone may foment excess glucocorticoid exposure in mPFC, as reviewed above. The combination of increased glucocorticoid exposure and elevated GABA has been hypothesized to be a combination that may lead to increased excitotoxicity in these regions, thereby partially explaining the structural alterations in these areas summarized in the preceding section. To date, however, the number of studies focused on GABA is relatively small, making it difficult to draw firm conclusions.

##### Imaging neuroendocrine and neuroimmune systems in MDD

Lastly, there has been growing interest in using neuroimaging to study the functional and structural consequence of other neurochemicals, such as hormones, factors and other endogenous signaling molecules. While technical limitations generally prevent the imaging of such molecules directly, their effects on structure and metabolism can nevertheless be observed using MRI. In the case of MDD, this work has focused most heavily on pro-inflammatory factors, including families of cytokines such as interleukins and interferons, hormones such as glucocorticoids (cortisol), thyroid stimulating hormone (TSH), and ghrelin [117,118].

Dysregulation of stress hormones has been widely reported in MDD (albeit with significant variance), and is believed to partially mediate some of the observed structural alterations associated with the disorder, especially within the hippocampus and mPFC [119,120]. These regions are well known for playing a critical role in the regulation of stress hormones via direct and indirect projections to the hypothalamus, and have been shown to be structurally vulnerable to glucocorticoid-mediated excitotoxicity. Animal studies using either chronic stress protocols or local corticosteroid injections have repeatedly demonstrated tissue damage following excessive glucocorticoid exposure, including loss of dendritic spines and de-arborization [121-124]. In human studies, comparable relationships have been observed between daily cortisol levels and grey-matter volume in depressed patients [125]. Given that elevated stress is a major precipitant of first-time depressive episodes [60], the association between stress and regional microdamage is highly relevant.

In addition to stress hormones, depressive states have been strongly associated with an elevated inflammatory load [126,127], and there has been growing interest in signaling pathways related to the metabolic syndrome and excess adipose tissue as potential mediators of chronic low-grade inflammation [128,129]. Neuroimaging has therefore been employed as an aid to understanding the possible consequences of altered inflammatory and metabolic factors on neural systems [130,131]. Animal models suggest that elevations of peripheral cytokines and subsequent CNS microglia activation can disrupt the synthesis of both 5-HT and DA [132,133], and induce symptoms of fatigue and motivational anhedonia [134,135]. While direct evidence of increased microglia activity in MDD has not been detected using available PET ligands [136], functional neuroimaging studies have sought to better understand the downstream consequences of increased cytokine activity by examining correlations between peripheral cytokine levels and resting or task-induced fluctuations in Blood-Oxygen-Level Dependent (BOLD) signals. Of note, healthy controls receiving an endotoxin challenge exhibited blunted neural responses to reward anticipation in the ventral striatum during a Monetary Incentive Delay (MID) task [137], similar to what is observed in MDD [42]. Further evidence comes from imaging studies of patients receiving IFN-α therapy. Following IFN-α treatment - which robustly increases inflammation - subjects exhibited decreased DA turnover within the striatum, as measured using a pre/post PET imaging of DA uptake with [^18^ F]Dopa (F-DOPA) [50]. By beginning to localize the functional architecture of brain regions that are affected by stress hormones and inflammation and by linking such abnormalities to specific cardinal symptoms of MDD (for example, anhedonia), these studies are playing a critical role in advancing a more precise understanding of depression symptomatology.

##### Summary of neuroimaging studies

In review of the findings above, several themes emerge. The most promising result is that regardless of imaging modality, neuroimaging studies repeatedly isolate a similar network of regions in which MDD patients differ from controls. Indeed, the greatest success of neuroimaging studies in MDD has been to identify core nodes involved in the expression of depressive symptoms. Neural responses in cortico-striato-limbic circuits have been shown to discriminate between responders to different treatment modalities [20,138] and have been the empirical foundation for new treatment techniques, such as deep brain stimulation (DBS) [139,140] and transcranial magnetic stimulation (TMS) [141].

But this is perhaps where the good news ends; for while the same regions are often implicated, the direction of the effects is often contradictory (for example, greater or lesser BOLD signal, depending on the task). Moreover, some of this consistency is undoubtedly due to confirmatory bias in ROI selection; that is, reports of group differences in a given region increase the probability that future studies will focus on the region, either with targeted measurement (for example, volumetric tracing) or with more liberal statistical thresholds in voxel-based studies (for example, small-volume correction). Even when group differences emerge, they are often present only at the level of group average, with comparable ranges for both groups (for example, [142]). As a result, the field has been unable to identify any neural signature that may serve as useful biomarker in the diagnosis of MDD, and guide treatment selection.

The lack of stable pathophysiological markers of MDD after so many neuroimaging studies raises the possibility that the biological origins of depressive symptoms are simply too dynamic to produce consistent patterns using case–control designs. For example, many receptor sub-types, including those of monoamine, Glu and GABA pathways show rapid, activity-dependent changes in expression and ligand affinity [143]. This plasticity may be affected by the time of day, the amount of sleep someone received two nights before, and how much they have been taxing their working memory in recent weeks just as much as it is affected by MDD ([105,143]). Against all these additional sources of variance, it is perhaps unsurprising that cross-sectional designs have been unable to identify reliable biomarkers.

As a result, an increasing number of researchers have called for a better understanding of specific circuits that may mediate transdiagnostic symptom expression [144,145]. While much of this discussion has rightly emphasized the importance of animal models [146,147], the final section presents several conceptual and methodological approaches to clinical imaging studies that we feel may aid in the identification of circuits rather than regions.

#### Future directions and circuit-based analysis

A clear limitation of current neuroimaging studies in MDD has been the tendency to assess particular measures of brain function independently, despite clear evidence that these measures are highly inter-related. In contrast to measures of specific brain regions, chemicals, or tissue classes, circuit-based analysis provides a conceptual framework that is organized around a specific behavioral process. Circuits are defined by the combined structural and functional properties that enable a specific set of computations to be performed on a discrete set of inputs. As such, circuit-analysis integrates across many different levels and measures of brain function, but eschew the complexity of simultaneous whole-brain analysis. By focusing on discrete computations with a circumscribed array of possible inputs and outputs, circuit analysis meets the basic scientific requirement of simplification, but does so without neglecting biological complexity.

**Figure 2:**
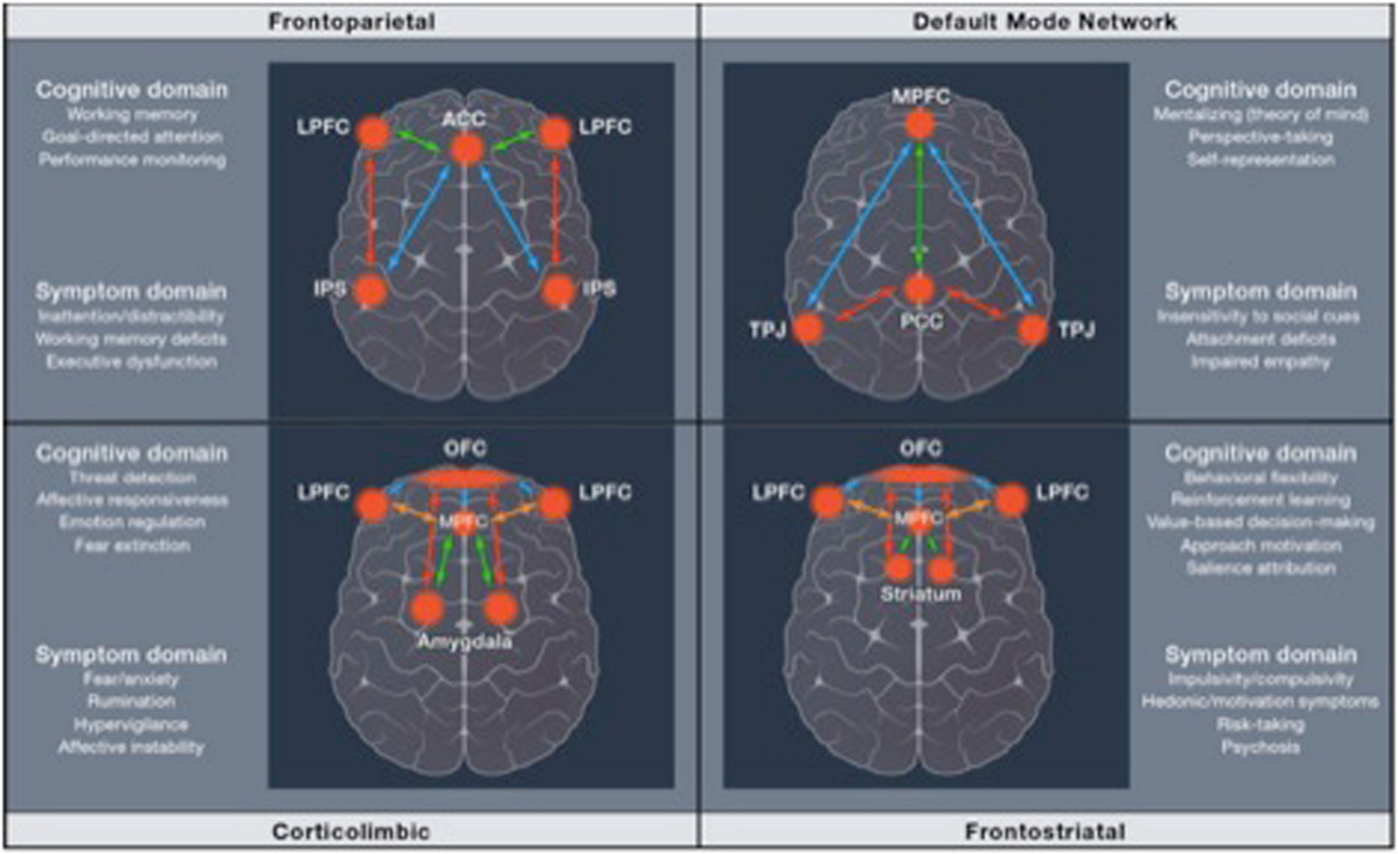
**Schematic depiction of commonly identified functional networks and their associated cognitive and symptom domains.** Republished with permission from Buckholtz and Meyer-Lindenberg [150].

One of the most powerful and readily available methods for circuit-analysis in patient populations is functional connectivity. Whereas much of the first two decades of neuroimaging research emphasized localization of function to a particular brain region while individuals were either at rest or performing a particular task, growing appreciation for the role of functional networks has emerged in recent years. This shift reflects a broader recognition in the field of neuroimaging that the brain is comprised of discrete networks, which show local properties (for example, 'small-worldness’) and can rapidly re-configure themselves to adapt to current environmental demands [148,149]. Consequently, focusing on task-driven changes within a single brain area provides only limited insight into the specific computations being performed. Indeed, significant progress has been made in the characterization of several stable networks that support domain-general functions, including attentional control, novelty and threat detection, default-mode and social cognition, and reinforcement learning and decision-making, all of which have been implicated in MDD [150] (Figure 2).

Complementing functional connectivity as a path towards understanding MDD from a systems-level approach is the growing use of multi-modal imaging. The most common example is the combined use of structural and function neuroimaging data. Such data are often collected together, as standard preprocessing pipelines for functional neuroimaging data typically require high-resolution structural scans [151]. More recently, the scope of multi-modal imaging studies has been expanded to include neurotransmitters such as monoamines, Glu and GABA. In an important study by Northoff and colleagues, researchers identified shifts in neurotransmitter availability of Glu and GABA as major drivers of altered functional responses to emotional stimuli in MDD [152]. Similarly, decreases in pre-synaptic striatal DA as measured using F-DOPA were associated with blunted ventral striatal BOLD signal during reward anticipation in depressed subjects, demonstrating a clear link between DA bioavailability and striatal responses to reward [50].

Another essential benefit of circuit-based analysis is the bridge it creates to behavioral and molecular neuroscience. Animal models play a pivotal role in testing causal hypotheses about brain function [146]. Such models are not only useful in aiding the interpretation of correlative neuroimaging data, they can also help refine the mapping of psychiatric symptom definitions to discrete and dissociable circuits. For example, the symptom of anhedonia - which was once perceived as a unitary construct tapping into the experience of pleasure - has gradually come to be recognized as involving multiple sub-components, including motivation and hedonic response, each of which involves distinct neural substrates [69,70,153]. However, because these sub-components in humans are often heavily correlated with each other at the level of symptom expression, animal models were required to demonstrate that motivational and hedonic aspects of reinforcement were indeed neurobiologically dissociable [48,67].

Pharmacological manipulations and other interventional techniques also provide tremendous advantages over imaging measures alone; these studies can identify plasticity dynamics that can help unpack some of the cross-sectional observations. For example, behavioral pharmacology studies of DA in MDD suggest that patients experience a much stronger euphoria response to amphetamine than controls [154,155], possibly indicating an up-regulation of post-synaptic DA receptors sites and/or DAT. The cross-sectional imaging studies discussed above, however, suggest down-regulation of both D1 and D2 as well as DAT proteins. Since the expression of these proteins is dynamic, imaging studies done in conjunction with pharmacological challenges (within-subject) hold the promise for stronger mechanistic inferences regarding how neurotransmitter systems are able to adapt to changing conditions in MDD. Such pharmacological challenges can therefore greatly aid in the understanding of plasticity within circuits, and help shift away from a focus on the identification of a stable, persistent pathological marker, which appears unlikely to exist.

Finally, circuit-based analysis may help facilitate a shift in the conceptualization and measurement of psychiatric symptom inventories that are often used to define groups and regress against imaging data. Psychiatric measurement largely relies on subjective self-reporting of distress as they *sine qua non* of symptom diagnosis. When it comes to reporting how one feels, however, studies in healthy populations have increasingly observed dissociation between the 'believing self’ and the 'experiencing self’ [156-158]. Accordingly, while the former describes how an individual summarizes their experience over a period of time, the latter refers to experiential reports made 'in the moment’. Whereas these constructs would theoretically be expected to correlate highly, growing evidence suggests that they are only moderately correlated at best [157,159]. This is in part due to the presence of well-known retrospective biases that reflect a heightened sensitivity to recency or maximum intensity of emotional experiences (so-called 'peak and end’ effects) [160]. In disorders like schizophrenia, such retrospective biases can result in almost completely uncorrelated findings of emotional response across retrospective and in-the-moment reports [161].

This renders significantly liable the common practice of regressing symptom severity measures (for example, the BDI-II) against imaging data [162], as both the independent and dependent measures likely reflect a complex mix of 'believing self’ and 'experiencing self’. For example, if someone has reported severe depression over the past week, but happens to have a brief lifting during the two- to three-hour window in which the lab experiment occurs, it may be more important to consider the 'in the moment’ affect rather than feelings aggregated over the past weeks when trying to interpret associated imaging data; this stands in contrast with the traditional assumption that individuals with shared symptom severity over a one-week period will have more variance in common than individuals with shared experience of a particular experimental task. One approach to addressing this challenge is the development of measures that seek to tease apart 'believing self’ and 'experiencing self’, with the aim of identifying separate biological correlates. It is likely that both are implicated in the maintenance of depressive symptoms [163], but current symptom assessment inventories are poorly suited to distinguish between these distinct modes of types of subjective report.

In sum, clinical imaging studies can contribute to circuit-based analysis through a focus on network-based analytical techniques, such as functional connectivity, multi-modal imaging methods, the use of within-subject pharmacological challenge designs, and greater sensitivity to potential discrepancies between 'believing self’ and 'experiencing self’ that may mask important distinctions in the relationships between subjective report and neuroimaging data.

## Conclusion

The neuroimaging literature of depression has grown tremendously over the last several decades. The primary fruit of these efforts has been the identification of brain regions and structures that are most critical to the expression of depressive symptomatology, while also increasing our knowledge of how these regions interact with particular neurotransmitter systems, neurochemicals, hormones, and other signaling proteins. Despite a wealth of positive findings, translations to treatment remain elusive. Moving forward, the integration of these various methods through the use of circuit-based analysis will be critical for the development of a biologically-based nosology and personalized medicine in psychiatry.

## Abbreviations

5-HT: Serotonin; ACC: anterior cingulate cortex; BOLD: blood-oxygen level-dependent; CNS: central nervous system; DA: dopamine; DAT: dopamine transporter; DBS: deep-brain stimulation; dlPFC: dorsolateral prefrontal cortex; Glu: glutamate; GABA: gamma-aminobutyric acid; HPA axis: hypothalamic-pituitary-adrenal axis; IFN: interferon; MDD: major depressive disorder; MID: monetary incentive delay; mPFC: medial prefrontal cortex; MRI: magnetic resonance imaging; MRS: magnetic resonance spectroscopy; NE: norepinephrine; NET: norepinephrine transporter; OFC: orbitofrontal cortex; PET: positron emission tomography; PFC: prefrontal cortex; ROI: region of interest; SPECT: single photon emission computed tomography; SERT: serotonin transporter; TMS: transcranial magnetic stimulation; TSH: thyroid stimulating hormone; VBM: voxel-based morphometry; vlPFC: ventrolateral prefrontal cortex.

## Competing interests

The authors declare no competing interests. Over the past three years, Dr. Pizzagalli received consulting/honoraria from AstraZeneca, Ono Pharma USA, Pfizer, Servier, and Shire for activities unrelated to the current review.

## Authors’ contributions

MTT and DAP developed the outline, MTT reviewed the relevant literature, and MTT and DAP wrote the manuscript. Both authors read and approved the final manuscript.
